# Effects of the juçara fruit (*Euterpe edulis* Martius) pulp and lyophilized extract on *NRF2*, *KEAP1*, *SOD1*, and *GPX2* expression in human colorectal cancer cell lines

**DOI:** 10.1590/1414-431X2023e12558

**Published:** 2023-04-14

**Authors:** L.A. Milholli, J. Dalbó, C.V.M.S. Couto, M.M. Oliveira, J.G. dos Santos, G.T. Peterle, A.B. Archanjo, P.I. Silva, J.N. Boeloni, F.D. Nunes, A.M.Á. da Silva, L.O. Trivilin

**Affiliations:** 1 Universidade Federal do Espírito Santo Departamento de Medicina Veterinária Centro de Ciências Agrárias e Engenharia Alegre ES Brasil Departamento de Medicina Veterinária, Centro de Ciências Agrárias e Engenharia, Universidade Federal do Espírito Santo, Alegre, ES, Brasil; 2 Universidade Federal do Espírito Santo Biotecnologia/Renorbio Programa de Pós-Graduação Alegre ES Brasil Biotecnologia/Renorbio Programa de Pós-Graduação, Universidade Federal do Espírito Santo, Alegre, ES, Brasil; 3 Universidade Federal do Espírito Santo Departamento de Engenharia de Alimentos Centro de Ciências Agrárias e Engenharias Alegre ES Brasil Departamento de Engenharia de Alimentos, Centro de Ciências Agrárias e Engenharias, Universidade Federal do Espírito Santo, Alegre, ES, Brasil; 4 Universidade de São Paulo Departamento de Estomatologia Faculdade de Odontologia São Paulo SP Brasil Departamento de Estomatologia, Faculdade de Odontologia, Universidade de São Paulo, São Paulo, SP, Brasil

**Keywords:** Anthocyanins, Antioxidants, Cytoprotection, Gene expression, Phenolic compounds

## Abstract

We investigated the effects of the juçara fruit (*Euterpe edulis* Martius) pulp and lyophilized extract on the expression of cytoprotective genes nuclear factor erythroid 2 (NF-E2)-related factor 2 (*NRF2*), kelch-like ECH-associated protein 1 (*KEAP1*), superoxide dismutase (*SOD1*), and glutathione peroxidase (*GPX2*) in human colorectal cancer cell lines (HT-29 and Caco-2). Cells were cultured for 24 h in Dulbecco's Modified Eagle's Medium containing juçara fruit pulp (5, 10, or 50 mg/mL) or lyophilized extract (0.05, 0.1, or 0.5 mg/mL), and gene expression was quantified using real-time quantitative reverse transcription polymerase chain reaction. All studied genes showed significant variation in gene expression among different concentrations of pulp or lyophilized extract. Overall, the expression of the selected genes decreased in both cell lines following exposure to the pulp or lyophilized extract in a dose-dependent manner for most of the concentrations studied. In summary, our study showed that the compounds in juçara fruit inhibited the expression of cytoprotective genes associated with the antioxidant response and that, although not cytotoxic at the concentrations studied, they could potentially block the activation of the *NRF2/KEAP1* pathway.

## Introduction

Colorectal cancer (CRC) is a chronic disease with a high rate of morbidity and mortality worldwide, and therefore a serious public health problem. According to the World Health Organization (WHO), CRC is the third most common and the second most lethal cancer in the world, with 2 million cases and 1 million deaths per year (1).

Etiological and epidemiological studies have attributed the increased incidence of CRC to several environmental factors, including consumption of red meat, smoking, and exposure of the intestinal tissue to carcinogens such as N-nitrous compounds, heterocyclic amines, and infectious agents (2).

The intestinal mucosa is naturally exposed to oxidizing and carcinogenic substances that induce the production of free radicals. The constant production of free radicals results in tissue and cellular damage and, consequently, chronic inflammation. The hydroperoxides contribute with the inflammatory microenvironment and severe oxidative stress can further damage normal cells. Overall, these events can stimulate carcinogenesis (3).

The antioxidant defense system of the body consists of several molecules that antagonize free radicals to reduce oxidative stress and protect cells from damage, including the enzymes superoxide dismutase (SOD), glutathione peroxidase (GPx), glutathione reductase, and catalase. Some vitamins, such as vitamin C and vitamin E, as well as phenolic compounds found in food also have antioxidant activity (4,5).

The phenolic compounds present in seeds, cereals, herbs, spices, roots, leaves, vegetables, and fruits represent a broad class of antioxidants. These antioxidants can inactivate reactive oxygen species and thereby affect several metabolic and molecular processes associated with neoplastic development (6). Thus, polyphenols act as protective agents by reducing oxidative damage, and are associated with the prevention of chronic diseases such as cancer (7).

The juçara fruit (*Euterpe edulis* Martius) stands out as a rich source of antioxidant compounds like anthocyanins, which can regulate the expression of cytoprotective genes such as *SOD1* and *GPx* (8). Therefore, we examined the effects of the juçara fruit pulp and lyophilized extract on the expression of cytoprotective genes *NRF2*, *KEAP1*, *SOD1*, and *GPX2* in human CRC cell lines.

## Material and Methods

### Preparation of the juçara fruit pulp and extraction of phenolic compounds

For this experiment, a single batch of juçara fruit pulp was obtained from a commercial supplier, Comércio e Beneficiamento de Polpas de Açaí EIRELI ME, Brazil).The pulp was free of dyes and preservatives and hermetically stored at -20°C for protection against light and atmospheric oxygen.

The extraction of phenolic compounds and anthocyanin pigments from the juçara fruit pulp was carried out using a previously published method (9). Briefly, 20 g of juçara fruit pulp was diluted in 200 mL of 70% cereal alcohol and the pH was adjusted to 2.5 with citric acid. The extract was then stored for 2 h at 4°C and protected from light and atmospheric oxygen. After that, the extract was filtered, concentrated in a rotary evaporator, and lyophilized. Lyophilized samples were hermetically stored at 4°C and protected from light until use.

Total anthocyanin content was quantified by a spectrophotometry-based pH-differential method (10) using potassium chloride buffer (0.025 M, pH 1.0) and sodium acetate buffer (0.4 M, pH 4.5). The anthocyanin-enriched extracts were diluted in each buffer (1:10 and 1:25, respectively), and absorbance was recorded at 510 and 700 nm wavelengths. Total anthocyanin content was expressed in milligrams of cyanidin-3-glucoside per gram of pulp (Cy-3-glu: molar mass: 449.2 g/mol, molar absorptivity: 26900 L·mol^-1^·cm^-1^). Thus, the average concentration of cyanidin-3-glucoside was about 107.08 mg per gram of pulp.

### Cell culture

Two human CRC cell lines, HT-29 and Caco-2 (Cell Bank, Brazil), were used. The cells were cultivated in Dulbecco's Modified Eagle's Medium (DMEM, Gibco, Germany). For the Caco-2 cell line, the medium was supplemented with 20% fetal bovine serum and 0.1% antibiotic-antimycotic solution. For HT-29 cells, the medium was supplemented with 10% fetal bovine serum and 1% antibiotic-antimycotic solution. Cell growth was monitored and replaced with fresh medium every two days until the cells reached at least 80% confluence.

### Cell viability and proliferation following exposure to juçara fruit pulp and lyophilized extract

For this analysis, 8×10^3^ HT-29 and Caco-2 cells were seeded onto 96-well plates and cultured for 48 h before exposure to the juçara fruit product. After that, medium containing different concentrations of the juçara fruit pulp (5, 10, and 50 mg/mL) and lyophilized extract (0.05, 0.1, and 0.5 mg/mL) was added to the culture, and cytostatic or cytotoxic effects were determined after 24 h. Control cells, unexposed to the juçara fruit product, were cultivated under the same conditions. The doses used were calculated such that the same amount of cyanidin-3-glucoside was present in both the pulp and extract.

Cell viability and proliferation were measured using the CellTiter 96^®^ AQueous One Solution Cell Proliferation Assay (Promega, USA). Briefly, exposed and unexposed cells were added to a 96-well plate, and 20 µL of the MTS substrate was added for each 100 µL of medium followed by incubation for 4 h at 37°C. Subsequently, the absorbance of each well was measured at 490 nm with a microplate reader (ELx800^TM^, BioTek Instruments, USA). All analyses were performed in triplicate, and the mean values were used for comparisons.

### RT-qPCR assay

For this assay, 1×10^5^ HT-29 and Caco-2 cells were seeded onto 6-well plates and cultured until they reached the ideal confluence. The medium containing different concentrations of the juçara fruit pulp (5, 10, and 50 mg/mL) and lyophilized extract (0.05, 0.1, and 0.5 mg/mL) was added, and total mRNA was extracted after 24 h.

Briefly, samples were incubated with 1.5 mL of Trizol (Invitrogen, USA) and gene expression was measured using GoTaq^®^ 1-Step RT-qPCR System (Promega A6020 kit). The analysis was performed on a 7500 Fast Real-Time PCR System (Applied Biosystems Inc., USA). All analyses were performed in triplicate. The relative expression of each gene was normalized to that of glyceraldehyde-3-phosphate dehydrogenase (*GAPDH*) using the 2^-ΔΔCT^ method. All real-time (RT) primers are listed in Table 1.

**Table 1:** List of primers used for RT-qPCR.

Primer name	Primer sequence
*SOD-1*	F: 5′-GCAGGTCCTCACTTTAATCCTC-3′
	R: 5′-AATAGACACATCGGCCACAC-3′
*NRF2*	F: 5′-CCGGCATTTCACTAAACACAAG-3′
	R: 5′-CAGAATCACTGAGGCCAAGTAG-3′
*KEAP1*	F: 5′-TGTCCTCAATCGTCTCCTTTATG-3′
	R: 5′-ACTCGTTCCTCTCTGGGTAG-3′
*GPX2*	F: 5′-CTTCTATGACCTCAGTGCCATC-3′
	R: 5′-TCAGAGCGAAGCCACATTC-3′
*GAPDH*	F: 5′-GGTGTGAACCATGAGAAGTATGA-3′
	R: 5′-GAGTCCTTCCACGATACCAAAG-3′

### Statistical analysis

GraphPad^®^ v.7.00 software (GraphPad Software, USA) was used for statistical analysis. Differences were considered significant at P<0.05. Means±SD were calculated for replicates. The cytostatic or cytotoxic effects were evaluated using two-way ANOVA followed by *post hoc* multiple comparisons *t*-tests. Relative expression levels of *NRF2*, *KEAP1*, *SOD1*, and *GPX2* were analyzed by one-way ANOVA followed by Tukey's test. The comparison of relative expression between cell lines at the different concentrations tested was assessed by multiple *t*-tests.

## Results

### Cell viability

**Figure 1:**
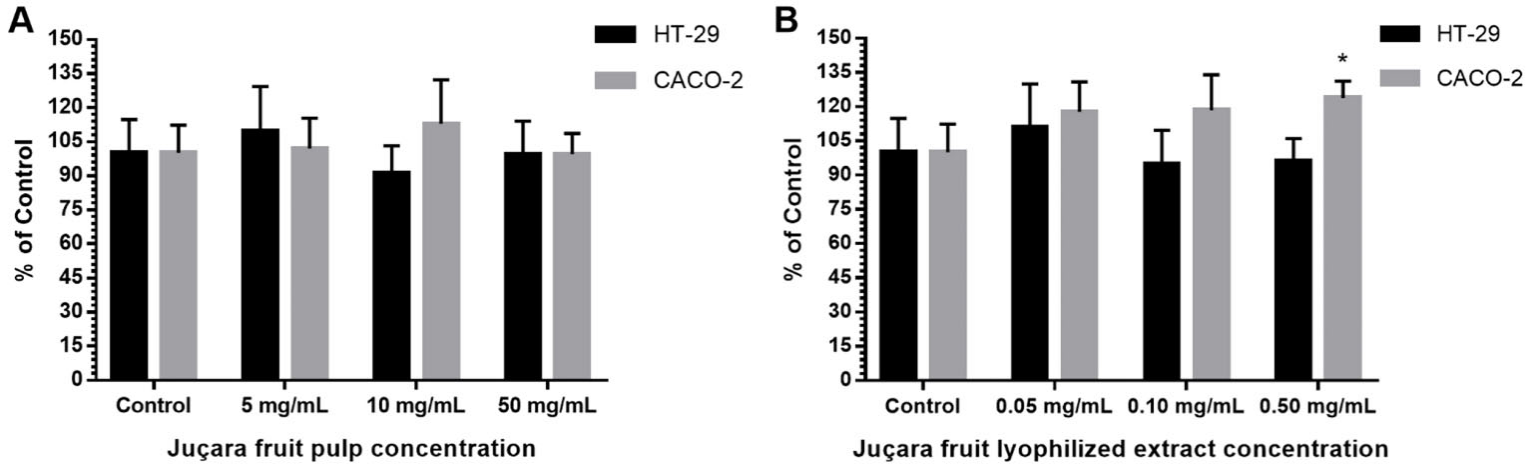
Cell viability and proliferation in Caco-2 and HT-29 cells exposed to different concentrations of pulp (**A**) and lyophilized extract (**B**) of the juçara fruit for 24 h. Cell viability was measured by the MTS assay. All reactions were prepared in triplicate and data are reported as means±SD. *P<0.05 compared to HT-29 as determined by multiple *t*-tests.

Juçara fruit pulp did not show any significant effect on cell viability (P=0.8734) or proliferation at the concentrations studied. Cell proliferation was also not affected and was similar in HT-29 and Caco-2 cell lines (P=0.5603) in the presence of the pulp (Figure 1A). Furthermore, the lyophilized extract of juçara fruit did not have any effect on cell viability (P=0.3596) at the concentrations studied. However, cell proliferation was significantly different between Caco-2 and HT-29 cells, particularly in the presence of 0.5 mg/mL extract (P=0.0204) (Figure 1B).

### Effect of juçara fruit pulp on *NRF2, KEAP1, SOD1*, and *GPX2* expression in HT-29 and Caco-2 cell lines

**Figure 2:**
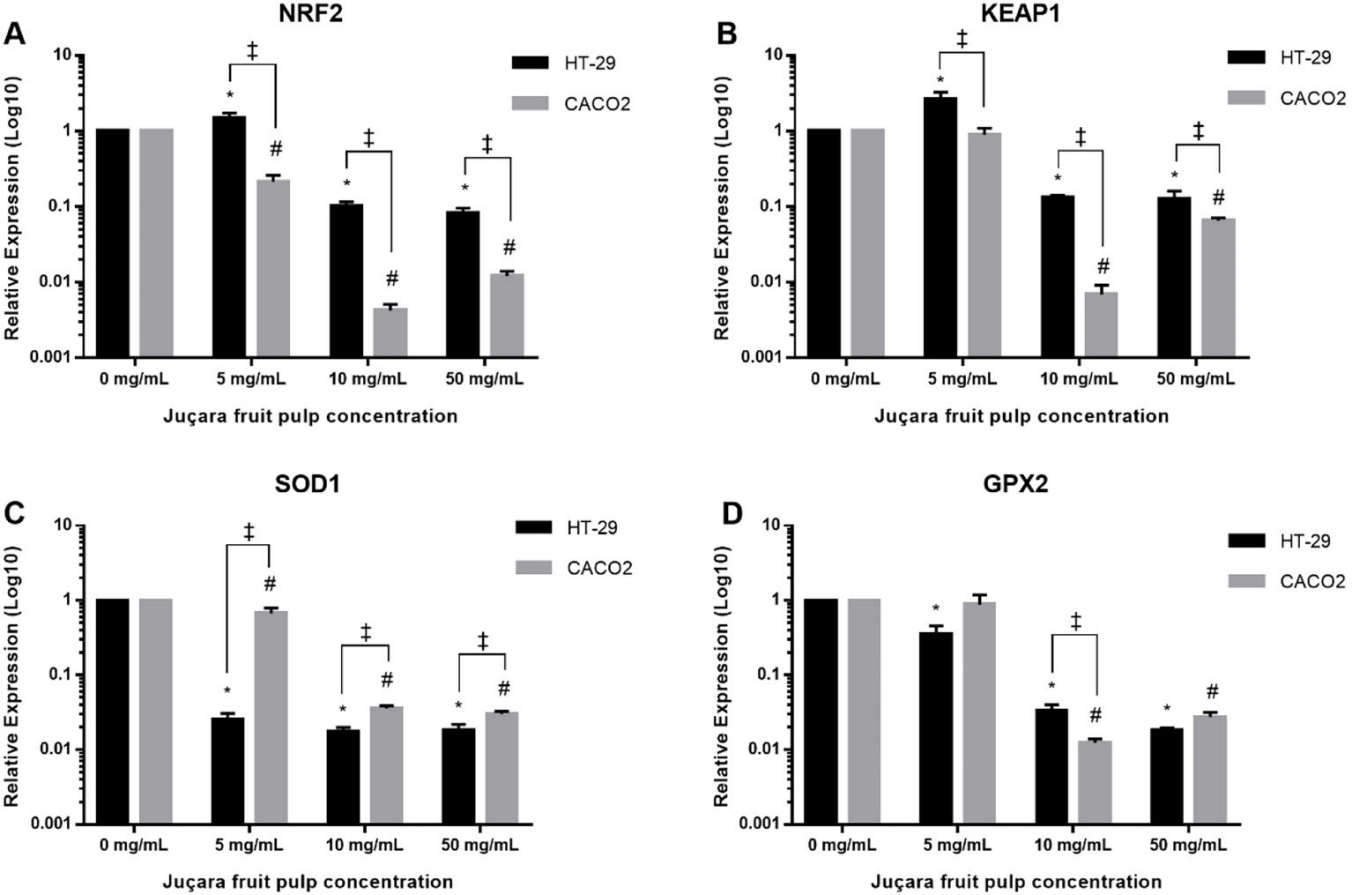
Relative expression of *NRF2*, *KEAP1*, *SOD1*, and *GPX2* genes in HT-29 and Caco-2 cell lines exposed to different concentrations of juçara fruit pulp for 24 h. All measurements were carried out in triplicate and the data are reported as means±SD. Gene expression was measured by RT-qPCR. *P<0.05 (HT-29) and ^#^P<0.05 (Caco-2) as determined by one-way ANOVA and *post hoc* Tukey's test; ^‡^P<0.05 as determined by multiple *t*-tests.

*NRF2* expression differed significantly when HT-29 cells were exposed to different juçara fruit pulp concentrations (P<0.0001), and it was higher in the lowest pulp concentration (5 mg/mL) than in the unexposed cells (P=0.0154) and in cells exposed to 10 mg/mL (P<0.0001) and 50 mg/mL (P<0.0001). For Caco-2 cells, *NRF2* expression also differed significantly in different juçara fruit pulp concentrations (P<0.0001) and was lower in the groups exposed. However, Caco-2 cells exposed to the 5 mg/mL pulp concentration showed greater expression compared to cells exposed to 10 mg/mL (P<0.0001) and 50 mg/mL (P<0.0001). It was observed that *NRF2* expression differed significantly between cell lines at 5 mg/mL (P=0.0015), 10 mg/mL (P=0.0005), and 50 mg/mL (P=0.0008) (Figure 2A).

Juçara fruit pulp also influenced *KEAP1* expression in HT-29 cells (P<0.0001). However, a greater expression was observed at a lower concentration (5 mg/mL) than in the unexposed group (P=0.0005) and in cells exposed to 10 mg/mL (P<0.0001) and 50 mg/mL (P<0.0001). For the Caco-2 cell line, juçara fruit pulp influenced *KEAP1* expression (P<0.0001) and was significantly lower in cells exposed to 10 and 50 mg/mL (P<0.0001). At 5 mg/mL, cells showed greater expression even when Caco-2 cells were not exposed (P=0.5606). When comparing the *KEAP1* expression between cell lines, HT-29 showed the highest expression at 5 mg/mL (P=0.0077), 10 mg/mL (P<0.0001), and 50 mg/mL (P=0.0290) (Figure 2B).

For *SOD1* expression in the HT-29 cell line, exposure to juçara fruit pulp promoted reduced expression at all concentrations used compared to unexposed cells (P<0.0001). However, no difference in *SOD1* expression was observed in cells exposed at 5, 10, and 50 mg/mL. In the Caco-2 cell line, pulp exposure significantly reduced *SOD1* expression (P<0.0001); however, cells exposed to 5 mg/mL pulp showed greater expression compared to cells exposed to 10 mg/mL (P<0.0001) and 50 mg/mL (P<0.0001). It was found that *SOD1* expression differed significantly between cell lines at 5 mg/mL (P=0.0008), 10 mg/mL (P=0.0012), and 50 mg/mL (P=0.0097) (Figure 2C).

Juçara fruit pulp altered *GPX2* expression in HT-29 cells (P<0.0001). At all concentrations tested, *GPX2* was under-expressed in relation to unexposed cells, but cells exposed to 5 mg/mL pulp showed higher expression compared to cells exposed to 10 mg/mL (P=0.0008) and 50 mg/mL (P=0.0012). In Caco-2 cells, *GPX2* expression also differed after juçara fruit pulp exposure (P=0.0003), but the expression was significantly lower in cells exposed to 10 mg/mL (P=0.0015) and 50 mg/mL (P=0.0016). Exposure to 5 mg/mL showed *GPX2* expression similar to that in unexposed cells (P=0.8434). Further, while comparing cell lines, *GPX2* expression was higher (P=0.0068) only in the HT-29 cells exposed to 10 mg/mL fruit pulp (Figure 2D).

### Effect of lyophilized juçara fruit extract on *NRF2, KEAP1, SOD1*, and *GPX2* expression in HT-29 and Caco-2 cell lines

**Figure 3:**
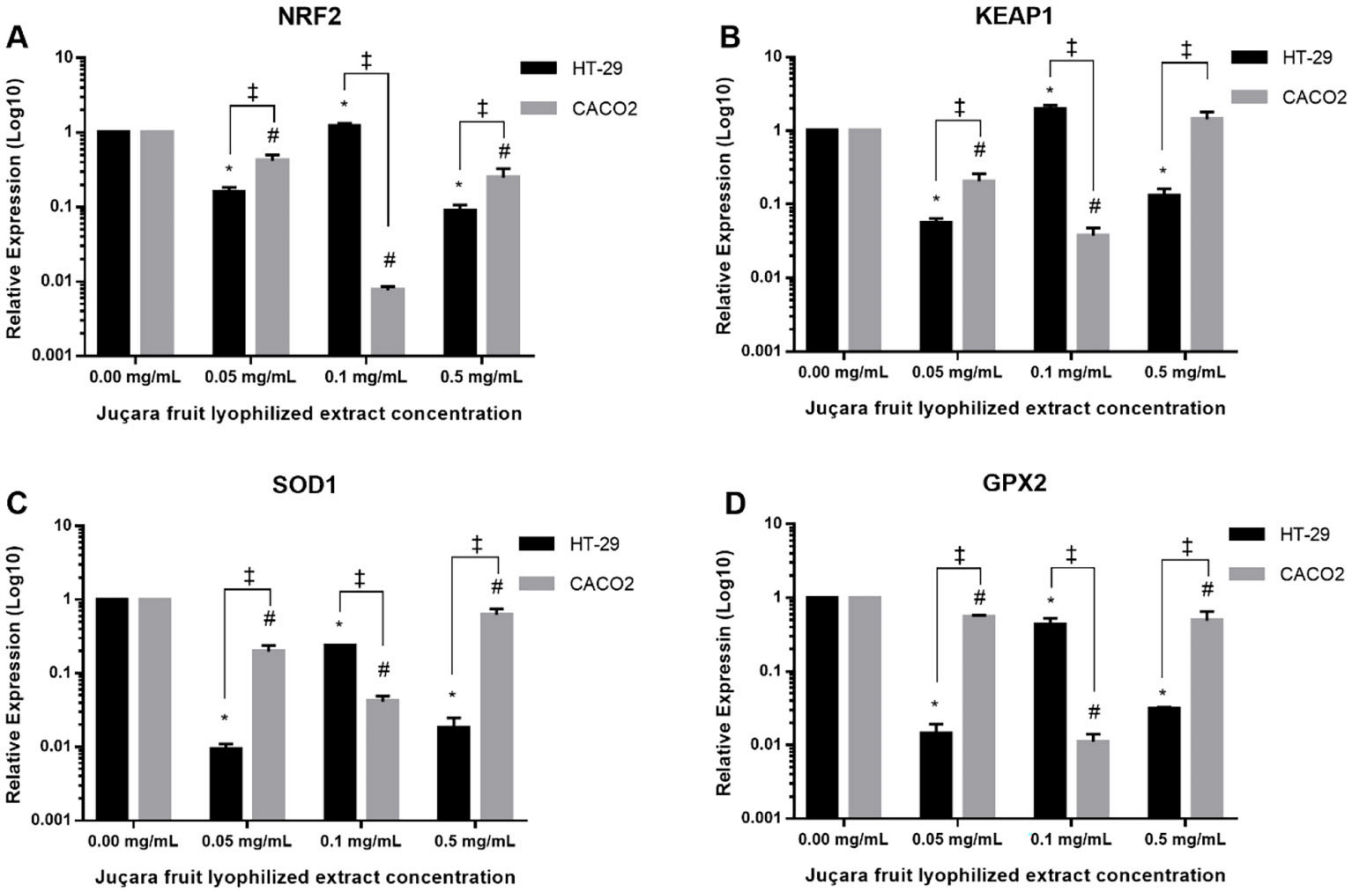
Relative expression of *NRF2*, *KEAP1*, *SOD1*, and *GPX2* in HT-29 and Caco-2 cell lines exposed to different concentrations of juçara fruit lyophilized extract for 24 h. All measurements were carried out in triplicate and the data are reported as means±SD. Gene expression was measured by RT-qPCR. *P<0.05 (HT-29) and ^#^P<0.05 (Caco-2) as determined by one-way ANOVA and *post hoc* Tukey's test; ^‡^P<0.05 as determined by multiple *t*-tests.

In HT-29 cells, *NRF2* expression differed significantly in different concentrations of juçara fruit lyophilized extract (P<0.0001), with the highest expression in cells exposed to 0.1 mg/mL (P=0.0172) and was under-expressed in cells exposed to 0.05 mg/mL (P<0.0001) and 0.5 mg/mL (P<0.0001) compared to unexposed cells. In the Caco-2 cell line, *NRF2* expression differed significantly in the different concentrations of extract (P<0.0001). *NRF2* under-expression was observed in all groups exposed to lyophilized extract; however, cells exposed to 0.05 mg/mL had a greater expression compared to other concentrations. When comparing *NRF2* expression between the two cell lines, Caco-2 cells demonstrated higher expression at 0.05 mg/mL and 0.5 mg/mL concentrations (P=0.0072 and P=0.0265, respectively), whereas at 0.1 mg/mL, HT-29 cells showed higher expression (P<0.0001) (Figure 3A).

Further, in the HT-29 cell line, exposure to juçara fruit lyophilized extract influenced *KEAP1* expression (P<0.0001). Compared to non-exposed cells, cells exposed to 0.1 mg/mL extract showed significantly higher expression (P=0.0004), whereas cells exposed to 0.05 and 0.5 mg/mL concentrations showed under-expression (P=0.0003 and P=0.0006, respectively). For Caco-2 cells, juçara fruit lyophilized extract also influenced *KEAP-1* expression (P<0.0001), and was lower at 0.05 mg/mL (P=0.0039) and 0.1 mg/mL (P=0.0011) compared to non-exposed cells. At 0.5 mg/mL, the expression was similar to that in the control group. Comparing HT-29 and Caco-2 cell lines, *KEAP1* expression was higher in Caco-2 at 0.05 mg/mL (P=0.0091) and 0.5 mg/mL (P=0.004). In contrast, at 0.1 mg/mL, HT- 29 showed greater expression (P=0.0004) (Figure 3B).

The effect of the juçara fruit lyophilized extract on *SOD1* expression in HT-29 cells showed that different concentrations promoted under-expression compared to the unexposed cells (P<0.0001). In the Caco-2 cell line, there was also a significant decrease in *SOD1* expression at all concentrations used compared to the unexposed cells (P<0.0001). When comparing the expression of *SOD1* between cell lines, HT-29 cells presented higher expression at 0.1 mg/mL (P<0.0001), while Caco-2 cells showed higher expression at 0.05 mg/mL (P=0.0035) and 0.5 mg/mL (P=0.0013) (Figure 3C).

*GPX2* expression differed significantly when HT-29 cells were exposed to different concentrations of juçara fruit lyophilized extract (P<0.0001) and was under-expressed compared to unexposed cells (P<0.0001). For Caco-2 cells, *GPX2* expression also differed significantly at different concentrations of juçara fruit lyophilized extract (P<0.0001) and was lower at 0.05 mg/mL (P=0.0039), 0.1 mg/mL (P<0.0001), and 0.5 mg/mL (P=0.0018) compared to unexposed cells. Further, when comparing the *GPX2* expression between the two cell lines, Caco-2 cells showed higher expression at 0.05 and 0.5 mg/mL (P<0.0001 and P=0.0340, respectively), while HT-29 showed greater expression (P=0.0015) at 0.1 mg/mL (Figure 3D).

## Discussion

In this study, both the pulp as well as the lyophilized extract that are rich in phenolic compounds, mainly anthocyanins, could not inhibit cell proliferation in the two cell lines studied. The anthocyanin (cyanidin-3-glucoside) did not affect HT-29 cell viability in another study as well. Anthocyanins, including cyanidin-3-glucoside, from grape skins did not inhibit the proliferation of a human colon cancer cell line (HCT-116) at a concentration of 200 μg/mL (11).

It is important to note that anthocyanin extracts from different sources vary in their structure and characteristics, such as glycosylation, and therefore have distinct anti-cancer functions (12). For instance, in contrast to non-acylated anthocyanin compounds from blueberry (*Vaccinium myrtillus* L.), anthocyanin-rich extracts containing acylated monoglycosides from grapes (*Vitis vinifera*) inhibited the proliferation of HT-29 cells (13). Moreover, another study showed that anthocyanins present in the acidified extract from blue corn (*Zea mays* L.) have antiproliferative activity in Caco-2 cell lines (14). Thus, antiproliferative effects are apparently dependent on the specific chemical characteristics of the anthocyanin present in the tested compound.

In addition to glycosylation, antiproliferative effects of anthocyanins are also dependent on variables such as period and type of storage (15). Further, the duration of exposure and anthocyanin concentrations also affect growth inhibition in HT-29 and Caco-2 cells (13,14). The concentrations and time of exposure to anthocyanins in juçara fruit products used in this study did not inhibit the proliferation of Caco-2 and HT-29 cell lines. However, the expression of cytoprotective genes responsible for redox state homeostasis and cell survival were modulated by the different concentrations of juçara fruit product studied.

Interestingly, *NRF2* expression was modulated in a dose-dependent manner in both human CRC cell lines used in this study. In addition, the *NRF2* expression in Caco-2 cells was lower than the expression in HT-29 cells following exposure to the juçara fruit pulp.

*NRF2* and CRC studies have shown that this protein is overexpressed in these neoplasms (16). Activation of the NRF2 pathway is a mechanism of the cellular antioxidant defense system to overcome redox imbalance and promote intestinal homeostasis (17). Thus, the *NRF2* under-expression observed in this study could make cancer cells more responsive to antineoplastic agents, considering that high *NRF2* expression is associated with HT-29 cells resistant to chemotherapeutic agents routinely used in CRC treatment (18).

Previous studies have shown that *NRF2* under-expression is helpful in CRC treatment as this protein provides cytoprotection against xenobiotics (19). Yokoo et al. (20) demonstrated that *NRF2* silencing prevented the increase in aberrant crypt foci and subsequent evolution into CRC by inhibition of the *COX2* gene. Notably, *NRF2* expression has been reported to offer resistance to CRC development (19). Thus, it can be hypothesized that under-expression of *NRF2* in cancer cells by the bioactive compound found in juçara fruit could cause cell death by lowering cytoprotection. In addition, a study that inhibited the expression of *NRF2* genes in HT-29 cells increased the cytotoxic activity of 5-fluorouracil, demonstrating that *NRF2* is a potential therapeutic target against CRC (21).

External factors, such as oxidative stress, directly induce *NRF2* expression. However, *NRF2* expression can also be induced indirectly by other mechanisms, such as signaling through the *KEAP1* repressor (22). According to Zimta et al. (23), miRNAs may play a role in *NRF2* regulation as miRNAs can inhibit the expression of cytoprotective genes including *KEAP1* and *NRF2*.

Moreover, anthocyanins possess inactive free radicals that can potentially affect the external factors involved in *NRF2* expression. The chemical structures of anthocyanins make them amenable to hydroxylation, such as the hydroxylation in the 3' position to form cyanidin-3-glucoside, thereby increasing their antioxidant capacity (24). The oxidation of anthocyanins helps in reducing reactive oxygen species (ROS)-induced cellular damage and oxidative stress (25). Neoplastic development is associated with ROS production and proliferation of intestinal stem cells (26). However, an exacerbated production of ROS can cause cell cycle arrest and apoptosis inhibition (27), this mechanism being used by cancer cells to obtain redox homeostasis (28).

NRF2/KEAP1 pathway inducers, such as ROS, interact with KEAP1 residues leading to a structural alteration of KEAP1 and a simultaneous release of NRF2, which enters the nucleus and induces the transcription of antioxidant enzymes (29).

Our results were similar to those of Kropat et al. (29) wherein anthocyanins from *Vaccinium myrtillus* L. and cyanidin-3-glucoside reduced the expression of *NRF2* and other cytoprotective genes in HT-29 cells. Moreover, Yan et al. (30) found that the anthocyanins present in blackberries decreased the expression of NRF2 in liver cells. Thus, anthocyanins present in the juçara fruit confer antioxidant protection and thereby reduce the expression of the genes studied in HT-29 and Caco-2 cells.

Overall, our analysis showed that *NRF2* expression is critical for cytoprotection and that modulation of its regulation by endogenous or exogenous agents could be used to make cancer cells more susceptible to treatment. Further studies are needed to understand the exact molecular mechanisms by which compounds in the juçara fruit regulate *NRF2* expression in CRC cell lines.

The expression of another important gene involved in cytoprotection, *KEAP1*, was also modulated in a dose-dependent manner by the juçara fruit product. Moreover, the pattern of *KEAP1* expression in HT-29 and Caco-2 cell lines was similar to that of *NRF2*. Shi et al. (31) observed that *KEAP1* under-expression in hepatocellular carcinoma is associated with overexpression of miR-141 and resistance of cancer cells to antineoplastic agents. Thus, it could be hypothesized that the compounds present in the juçara fruit decrease *KEAP1* and *NRF2* expression and, consequently, the expression of other antioxidant enzymes studied via mechanisms involving miRNAs.

The NRF2/KEAP1 signaling pathway is an important component of the antioxidant defense system. Skrzycki et al. (32) observed that in hypoxia, *SOD1* expression was reduced in human CRC cells, thus indicating low levels of ROS production and the absence of oxidative stress. In this study, *SOD1* expression was lower in HT-29 and Caco-2 cells exposed to the juçara fruit pulp and lyophilized extract than in control cell lines. In addition, *SOD1* expression levels were higher in Caco-2 than in HT-29 cells following exposure to the juçara fruit but not to the lyophilized extract. Our observations suggested that the compounds present in the juçara fruit could have neutralized ROS, thereby decreasing the oxidative stress and consequently reducing the expression of *SOD1*.

*SOD1* overexpression is a characteristic of CRC, and increased SOD1 and GPX2 activity, primarily due to induced gene expression, was observed in several patients with advanced stages of the disease (33). In addition, the increased activity of SOD1 results in a higher transformation rate of the superoxide radical, thus promoting the production of hydrogen peroxide, which is also associated with some types of human cancers (34). Further, free radicals induce the production of angiogenic factors that favor tumor growth and metastasis (35). Dos Reis et al. (8) showed that including the juçara fruit in the diet of carcinogen-induced mice decreased the number of aberrant crypts in the colon and rectum and increased SOD1 protein expression, thereby conferring protection to the colonic mucosa, which demonstrated the ability of phenolic compounds to reduce oxidative stress. Thus, the under-expression of *SOD1* observed in this study could be potentially due to the ability of compounds present in the juçara fruit to control oxidative stress without the need for induction of antioxidant enzymes, as shown by the low expression of *NRF2*. Additional studies are needed to understand the exact role of juçara fruit compounds in chemoprevention.

The expression of *GPX2* was similar to *SOD1*, and it was negatively modulated in a dose-dependent manner in the two human CRC cell lines used in this study. *GPX2* mRNA decreased as the concentration of the juçara fruit pulp in the culture medium increased. However, the same pattern was not observed in cells exposed to the lyophilized extract. Increased *GPX2* mRNA was observed at the median concentration (0.1 mg/mL) in HT-29 cells and at a 0.5 mg/mL in Caco-2 cells. In all the other studied concentrations, Caco-2 cells had higher levels of *GPX2* mRNA than HT-29 cells in the presence of juçara fruit compounds.

*GPX2* is involved in Wnt signaling and NRF2 signaling and contains promoter binding sites for the binding of associated transcription factors, such as β catenin/TCF and NRF2. Studies have shown that natural substances rich in flavonoids, such as sulfurane and curcumin, can activate the GPX2 transition via the NRF2 pathway (36). Emmink et al. (28) demonstrated that inhibition of GPX2 by the NRF2 pathway in human CRC cells makes these cells more susceptible to cell death and the action of antineoplastic agents, such as cisplatin, resulting in high levels of intracellular ROS and consequently apoptosis of these cells. In addition, they showed that GPX2 plays an important role in tumor development, such as the survival of neoplastic cells against ROS, favoring the growth of established tumors and promoting the development of neoplastic masses.

Thus, the *GPX2* under-expression observed in this study could be the consequence of reduced *NRF2* expression. Thus, the under-expression of *GPX2* following exposure to juçara fruit compounds may be useful in cancer therapy, based on the reduced antioxidant defense potential shown in HT-29 and Caco-2 cells. Notably, high expression of *GPX2* is useful in controlling inflammation by inhibiting COX2; however, in CRC, it increases the rate of cell proliferation and decreases apoptosis in cancer cells (36).

The differences in expression between the HT-29 and Caco-2 cell lines are explained by the fact that they are different neoplastic types. The HT-29 cell line is derived from a colorectal adenocarcinoma in a Caucasian adult female (44 years old) whereas the Caco-2 cell line is derived from a colorectal adenocarcinoma in a Caucasian adult male (77 years old) (37). The differences in gene expression could also be due to the cells being in different stages of neoplastic development and therefore respond differently to changes in the microenvironment and metabolism. Although differences in gene expression between the studied cell lines have been highlighted throughout the discussion, the overall trends were similar in that the expression of the studied genes decreased as the dose of the juçara fruit product increased.

In summary, the juçara fruit pulp or extract did not interfere in the proliferation of HT-29 and Caco-2, but modulated the expression of cytoprotective genes *NRF2*, *KEAP1*, *SOD1*, and *GPX2* in a dose-dependent manner, with a tendency to an under-expression of the NRF2/KEAP1 pathway at higher concentrations. Thus, the pulp and extract of the juçara fruit can potentially reduce the cytoprotection conferred by the genes associated with the antioxidant defense system in human CRC cells. The study also highlighted the pathway as a potential therapeutic target. Further studies are needed to elucidate the exact mechanisms underlying the activity of these products against neoplastic cells.

## References

[1] 1. Sung H, Ferlay J, Siegel RL, Laversanne M, Soerjomataram I, Jemal A, et al. Global cancer statistics 2020: GLOBOCAN estimates of incidence and mortality worldwide for 36 cancers in 185 countries. CA: A Cancer J Clin 2021; 71: 209-249, doi: 10.3322/caac.21660. 10.3322/caac.21660 33538338

[2] 2. Torre LA, Siegel RL, Ward EM, Jemal A. Global cancer incidence and mortality rates and trends - an update. Cancer Epidemiol Biomarkers Prev 2016; 25: 16-27, doi: 10.1158/1055-9965.EPI-15-0578. 10.1158/1055-9965.EPI-15-0578 26667886

[3] 3. Guz J, Foksinski M, Siomek A, Gackowski D, Rozalski R, Dziaman T, et al. The relationship between 8-oxo-7,8-dihydro-2'-deoxyguanosine level and extent of cytosine methylation in leukocytes DNA of healthy subjects and in patients with colon adenomas and carcinomas. Mut Res 2008; 640: 170-173, doi: 10.1016/j.mrfmmm.2007.12.013. 10.1016/j.mrfmmm.2007.12.013 18281064

[4] 4. Fukumura D, Kashiwagi S, Jain RK. The role of nitric oxide in tumour progression. Nat Rev Cancer 2006; 6: 521-534, doi: 10.1038/nrc1910. 10.1038/nrc1910 16794635

[5] 5. Peng C, Wang X, Chen J, Jiao R, Wang L, Li YM, et al. Biology of ageing and role of dietary antioxidants. Biomed Res Int 2014; 2014: 1-13. 10.1155/2014/831841 PMC3996317 24804252

[6] 6. Mazzola M, Carini F, Damiani P, Jurjus AR, Gerges A, Jurjus RA, et al. Inflammatory bowel disease and colorectal cancer, nutraceutical aspects. EuroMediterranean Biomed J 2016; 11: 123-129.

[7] 7. Wang LS, Kuo C, Cho S, Seguin C, Siddiqui J, Stoner K, et al. Black raspberry-derived anthocyanins demethylate tumor suppressor genes through the inhibition of DNMT1 and DNMT3B in colon cancer cells. Nutr Cancer 2013; 65: 118-125, doi: 10.1080/01635581.2013.741759. 10.1080/01635581.2013.741759 PMC3570951 23368921

[8] 8. Dos Reis SO, da Luz TC, Couto CVMS, Dalbó J, Nunes LC, Martins MC, et al. Juçara (*Euterpe edulis* Mart.) Supplementation reduces aberrant crypt foci and increases SOD1 expression in the colorectal mucosa of carcinogenesis-induced rats. Nutr Cancer 2020; 72: 610-619, doi: 10.1080/01635581.2019.1649437. 10.1080/01635581.2019.1649437 31441671

[9] 9. Francis FG. Analysis of anthocyanins. In: Anthocyanins as food colors (P. Markakis ed.). New York: Academic Press; 1982. p 182-208.

[10] 10. Giusti M, Wrolstad RE. Characterization and Measurement of Anthocyanins by UV Visible Spectroscopy. Curr Protoc Food Anal Chem 2001; F1.2.1-F1.2.13, doi: 10.1002/0471142913.faf0102s00.

[11] 11. Serra D, Paixão J, Nunes C, Dinis TCP, Almeida LM. Cyanidin-3-glucoside suppresses cytokine-induced inflammatory response in human intestinal cells: comparison with 5-aminosalicylic acid. PLoS One 2013; 8: e73001, doi: 10.1371/journal.pone.0073001. 10.1371/journal.pone.0073001 PMC3765207 24039842

[12] 12. Moraes LFS, Sun X, Peluzio MCG, Zhu MJ. Anthocyanins/anthocyanidins and colorectal cancer: what is behind the scenes? Critical Rev in Food Sci Nutr 2017; 59: 1-13, doi: 10.1080/10408398.2017.1357533. 10.1080/10408398.2017.1357533 28799785

[13] 13. Zhao C, Giusti M, Malik M, Moyer MP, Magnuson BA. Effects of commercial anthocyanin-rich extracts on colonic cancer and nontumorigenic colonic cell growth. J Agric Food Chem 2004; 52: 6122-6128, doi: 10.1021/jf049517a. 10.1021/jf049517a 15453676

[14] 14. Urias-Lugo DA, Heredia JB, Muy-Rangel MD, Valdez-Torres JB, Serna-saldívar SO, Gutierrez-Uribe JA. Anthocyanins and phenolic acids of hybrid and native blue maize (*Zea mays* L.) Extracts and their antiproliferative activity in mammary (MCF7), liver (HepG2), colon (Caco2 and HT29) and prostate (PC3) cancer cells. Plant Foods Hum Nutr 2015; 70: 193-199, doi: 10.1007/s11130-015-0479-4. 10.1007/s11130-015-0479-4 25762472

[15] 15. Blessington T, Nzaramba MN, Scheuring DC, Hale AL, Reddivari L, Miller C. Cooking methods and storage treatments of potato: effects on carotenoids, antioxidant activity, and phenolics. Am J Potato Res 2010; 87: 479-491, doi: 10.1007/s12230-010-9150-7.

[16] 16. Zhao XQ, Zhang YF, Xia YF, Zhou ZM, Cao YQ. Promoter demethylation of nuclear factor-erythroid 2-related factor 2 gene in drug-resistant colon cancer cells. Oncol Lett 2015; 10: 1287-1292, doi: 10.3892/ol.2015.3468. 10.3892/ol.2015.3468 PMC4533726 26622665

[17] 17. Kuo HCD, Wu R, Li S, Yang AY, Kong AN. Anthocyanin delphinidin prevents neoplastic transformation of mouse skin JB6 P+ cells: epigenetic re-activation of Nrf2-are pathway. AAPS J 2019; 21: 83-103, doi: 10.1208/s12248-019-0355-5. 10.1208/s12248-019-0355-5 PMC6669902 31254216

[18] 18. Kang KA, Piao MJ, Ryu YS, Kang HK, Chang WY, Keum YS, et al. Interaction of DNA demethylase and histone methyltransferase upregulates NRF2 in 5-fluorouracil-resistant colon cancer cells. Oncotarget 2016; 7: 40594-40620, doi: 10.18632/oncotarget.9745. 10.18632/oncotarget.9745 PMC5130030 27259240

[19] 19. Arlt A, Bauer I, Schafmayer C, Tepel J, Müerköster SS, Brosch M, et al. Increased proteasome subunit protein expression and proteasome activity in colon cancer relate to an enhanced activation of nuclear factor E2-related factor 2 (NRF2). Oncogene 2009; 28: 3983-3996, doi: 10.1038/onc.2009.264. 10.1038/onc.2009.264 19734940

[20] 20. Yokoo Y, Kijima A, Ishii Y, Takasu S, Tsuchiya T, Umemura T. Effects of NRF2 silencing on oxidative stress-associated intestinal carcinogenesis in mice. Cancer Med 2016; 5: 1228-1238, doi: 10.1002/cam4.672. 10.1002/cam4.672 PMC4924381 26899729

[21] 21. Akhdar H, Loyer P, Rauch C, Corlu A, Guillouzo A, Morel F. Involvement of Nrf2 activation in resistance to 5-fluorouracil in human colon cancer HT-29 cells. Eur J Cancer 2009; 45: 2219-2227, doi: 10.1016/j.ejca.2009.05.017. 10.1016/j.ejca.2009.05.017 19524433

[22] 22. Gonzalez-Donquiles C, Alonso-Molero J, Fernandez-Villa T, et al. The NRF2 transcription factor plays a dual role in colorectal cancer: a systematic review. PLoS One 2017; 12: e0177549, doi: 10.1371/journal.pone.0177549. 10.1371/journal.pone.0177549 PMC5436741 28542357

[23] 23. Zimta AA, Cenariu D, Irimie A, Magdo L, Nabavi SM, Atanasov AG, et al. The role of Nrf2 activity in cancer development and progression. Cancers (Basel) 2019; 11: 1755, doi: 10.3390/cancers11111755. 10.3390/cancers11111755 PMC6896028 31717324

[24] 24. Kuskoski EM, Asuero AG, Troncoso AM, Fett R. Actividad antioxidante de pigmentos antociánicos. Food Sci Technol 2004; 24: 691-693, doi: 10.1590/S0101-20612004000400036.

[25] 25. Nijveldt RJ, van Nood E, van Hoorn DE, Boelens PG, van Norren K, van Leeuwen PA. Flavonoids: a review of probable mechanisms of action and potenctial applications. Am J Clin Nutr 2001; 74: 418-425, doi: 10.1093/ajcn/74.4.418. 10.1093/ajcn/74.4.418 11566638

[26] 26. Myant KB, Cammareri P, McGhee EJ, Ridgway RA, Huels DJ, Cordero JB, et al. ROS production and NF-kappaB activation triggered by rac1 facilitate Wnt-driven intestinal stem cell proliferation and colorectal cancer initiation. Cell Stem Cell 2013; 12: 761-773, doi: 10.1016/j.stem.2013.04.006. 10.1016/j.stem.2013.04.006 PMC3690525 23665120

[27] 27. Nogueira V, Hay N. Molecular pathways: reactive oxygen species homeostasis in cancer cells and implications for cancer therapy. Clin Cancer Res 2013; 19: 4309-4314, doi: 10.1158/1078-0432.CCR-12-1424. 10.1158/1078-0432.CCR-12-1424 PMC3933310 23719265

[28] 28. Emmink BL, Laoukili J, Kipp AP, Koster J, Govaert KM, Fatrai S, et al. GPx2 suppression of H_2_O_2_ stress links the formation of differentiated tumor mass to metastatic capacity in colorectal cancer. Cancer Res 2014; 74: 6717-6730, doi: 10.1158/0008-5472.CAN-14-1645. 10.1158/0008-5472.CAN-14-1645 25261240

[29] 29. Kropat C, Mueller D, Boettler U, Zimmermann K, Heiss EH, Dirsch VM, et al. Modulation of Nrf2-dependent gene transcription by bilberry anthocyanins *in vivo*. Mol Nutr Food Res 2013; 57: 545-550, doi: 10.1002/mnfr.201200504. 10.1002/mnfr.201200504 23349102

[30] 30. Yan F, Chen X, Zheng X. Protective effect of mulberry fruit anthocyanin on human hepatocyte cells (LO2) and Caenorhabditis elegans under hyperglycemic conditions. Food Res Int 2017; 102: 213-224, doi: 10.1016/j.foodres.2017.10.009. 10.1016/j.foodres.2017.10.009 29195942

[31] 31. Shi L, Wu L, Chen Z, Yang J, Chen X, Yu F, et al. MiR-141 Activates Nrf2-dependent antioxidant pathway via down-regulating the expression of Keap1 conferring the resistance of hepatocellular carcinoma cells to 5-Fluorouracil. Cell Physiol Biochem 2015; 35: 2333-2348, doi: 10.1159/000374036. 10.1159/000374036 25896253

[32] 32. Skrzycki M, Czeczot H, Chrzanowska A, Otto-Ślusarczyk D. The level of superoxide dismutase expression in primary and metastatic colorectal cancer cells in hypoxia and tissue normoxia [in Polish]. Pol Merkur Lekarski 2015; 39: 281-286. 26637092

[33] 33. Skrzydlewska E, Sulkowski S, Koda M, Zalewski B, Kanczuga-Koda L, Sulkowska M. Lipid peroxidation and antioxidant status in colorectal cancer. World J Gastroenterol 2005; 11: 403-406, doi: 10.3748/wjg.v11.i3.403. 10.3748/wjg.v11.i3.403 PMC4205348 15637754

[34] 34. Szatrowski TP, Nathan CF. Production of large amounts of hydrogen peroxide by human tumor cells. Cancer Res 1991; 51: 794-798. 1846317

[35] 35. Brown NS, Jones A, Fujiyama C, Harris AL, Bicknell R. Thymidine phosphorylase induces carcinoma cell oxidative stress and promotes secretion of angiogenic factors. Cancer Res 2000; 60: 6298-6302. 11103787

[36] 36. Brigelius-Flohé R, Kipp AP. Physiological functions of GPx2 and its role in inflammation-triggered carcinogenesis. Ann NY Acad Sci 2012; 1259: 19-25, doi: 10.1111/j.1749-6632.2012.06574.x. 10.1111/j.1749-6632.2012.06574.x 22758632

[37] 37. American Type Culture Collection (ATCC) (2020) ATCC Guide to Packaging and Shipping of Biological Materials. American Type Collection Culture. USA, Jan. 2020. https://atcc.org/Products/All/HTB-38.aspx. Accessed January, 2021.

